# Differentiating Sepsis from Infectious and Non-Infectious Conditions in Patients with Elevated Procalcitonin Levels: Performance of Procalcitonin, CRP, and Lactate

**DOI:** 10.3390/jcm15166132

**Published:** 2026-08-07

**Authors:** Kamil Deveci, Necmi Baykan, Fatma Hançer Çelik, Fatma Ünlü, Esma Eryılmaz Eren, Davut Eren, Maruf Boran, Ömer Salt

**Affiliations:** 1 Department of Intensive Care, Kayseri City Education and Research Hospital, 38080 Kayseri, Türkiye; kamildeveci@gmail.com (K.D.); marufboran@gmail.com (M.B.); 2 Department of Emergency, Kayseri City Education and Research Hospital, 38080 Kayseri, Türkiye; drnecmibaykan@gmail.com (N.B.); fatmaunlu1995@gmail.com (F.Ü.); dromersalt@gmail.com (Ö.S.); 3 Department of Emergency, Gaziantep City Education and Research Hospital, 27470 Gaziantep, Türkiye; ftmhncr91f@gmail.com; 4 Department of Infectious Disease and Clinical Microbiology, Kayseri City Education and Research Hospital, University of Health Sciences, 38080 Kayseri, Türkiye; 5 Department of Nephrology, Kayseri City Education and Research Hospital, 38080 Kayseri, Türkiye; dr.davuteren@gmail.com

**Keywords:** sepsis, infectious diseases, biomarkers

## Abstract

**Background/Objectives**: Elevated Procalcitonin (PCT) levels are frequently encountered in clinical practice but may occur in both infectious and non-infectious conditions, making their interpretation challenging. This study evaluated the diagnostic performance of PCT, CRP, and lactate for differentiating sepsis, infectious conditions, and non-infectious conditions in patients with elevated baseline PCT levels. **Methods**: In this single-center retrospective observational study, 1072 adults presenting to the ED between June and December 2024 with serum PCT ≥ 0.51 ng/mL were included. Patients were classified into sepsis (*n* = 272), infection (*n* = 618), and non-infection (*n* = 182) groups according to the Sepsis-3 criteria and comprehensive clinical adjudication. ROC analyses and DeLong tests were performed to evaluate biomarker performance. **Results**: For identifying sepsis, PCT demonstrated better discriminatory performance (AUC 0.683) than CRP (AUC 0.619) and lactate (AUC 0.620), with significant differences between PCT and both CRP (*p* = 0.009) and lactate (*p* = 0.018). The optimal PCT cut-off was 2.35 ng/mL (sensitivity 65.8%, specificity 59.5%). For differentiating infectious from non-infectious conditions, CRP showed modest discrimination (AUC 0.640), whereas PCT (AUC 0.581) and lactate (AUC 0.439) performed poorly. Lactate showed the highest prognostic performance for in-hospital mortality (AUC 0.598), although overall prognostic accuracy remained limited. **Conclusions**: In ED patients with elevated baseline PCT levels, PCT showed better discrimination for sepsis than CRP and lactate, whereas CRP performed modestly in differentiating infectious from non-infectious conditions. None of the biomarkers demonstrated sufficient accuracy for standalone clinical use, supporting their role as complementary tools alongside comprehensive clinical assessment and the Sepsis-3 criteria.

## 1. Introduction

Sepsis is a critical clinical condition associated with high mortality rates and requiring early diagnosis. Since there is no gold standard for diagnosis, inflammatory markers and organ function scoring systems are used in the diagnostic approach [1,2,3]. White Blood Cell Count (WBC), C-reactive protein (CRP), Procalcitonin (PCT), and Lactate levels are the most commonly studied tests in this regard.

Procalcitonin (PCT) is the prohormone of calcitonin, encoded by the CALC-1/CALCA gene. It is found at very low levels in the bloodstream (<0.05 ng/mL) because it is converted to calcitonin, which plays a role in calcium homeostasis [4,5]. However, in the case of bacterial infection, various non-endocrine tissues also secrete PCT. In bacterial infections, it is released in higher concentrations in response to endotoxins and mediators released by the body. Serum PCT levels have been shown to rise within 2–4 h following stimulation by bacterial endotoxins, with a half-life of 24 to 36 h [2]. Today, Procalcitonin is used as a biomarker in the diagnosis and treatment monitoring of patients with bacterial infections, and in differentiating bacterial infections from other inflammatory conditions. Guidelines exist for PCT monitoring in the diagnosis of sepsis and in deciding on treatment continuation [6].

Lactate level is an important metabolic parameter reflecting tissue perfusion impairment and cellular hypoxia, and it has prognostic value in sepsis. However, Lactate’s performance in distinguishing sepsis from infection or other critical conditions may vary depending on the population. Lactate impacts immune cell function importantly in sepsis and septic shock, going beyond the traditional belief that it is simply a metabolic byproduct [7]. Therefore, evaluating the diagnostic contribution of PCT, CRP, and Lactate together is important for both strengthening early diagnosis and supporting clinical decision-making processes [8].

The aim of this study is to evaluate the diagnostic utility of PCT, CRP, and Lactate for distinguishing sepsis, infectious conditions, and non-infectious conditions in patients presenting to the emergency department with elevated PCT levels.

## 2. Materials and Methods

### 2.1. Study Design and Population

This single-center, retrospective observational study was conducted at Kayseri City Education and Research Hospital. A total of 1072 adult patients who presented to the emergency department between June and December 2024 and had a serum PCT level ≥ 0.51 ng/mL at admission were included in the study. During the study period, a total of 406,652 emergency department visits were recorded. These visits constituted the initial screening population from which eligible adult patients with serum PCT measurements were identified. This threshold corresponded to the institutional laboratory definition of an elevated PCT result during the study period, in which serum PCT values > 0.50 ng/mL were reported as ≥0.51 ng/mL. It was predefined before data collection, was not derived from the study data, and is consistent with the commonly accepted clinical threshold of 0.5 ng/mL recommended in current clinical practice guidelines [9]. The study was approved by the Clinical Research Ethics Committee of Kayseri City Education and Research Hospital (Decision No.: 310, Date: 28 January 2025). All procedures were conducted in accordance with the Declaration of Helsinki and applicable institutional and national ethical standards. To enhance transparency, this study was reported in accordance with the Strengthening the Reporting of Observational Studies in Epidemiology (STROBE) guidelines.

Patients’ demographic, clinical, and laboratory parameter data were obtained via our hospital’s electronic medical record system (KEYDATA). For all patients, the following data were recorded: Demographic variables: age, sex and comorbidities. Presentation-related variables: chief complaint at admission, disposition status (hospitalization or discharge), and need for intensive care unit (ICU). Laboratory parameters: complete blood count (white blood cell count, neutrophil count, hemoglobin, platelet count), biochemical markers (blood urea nitrogen, creatinine, AST, ALT), inflammatory markers (CRP, PCT), and Lactate level. All laboratory values were obtained via blood samples collected within the initial ED presentation.

This study aimed to evaluate whether CRP, Lactate, and varying degrees of PCT elevation could help differentiate sepsis, infection, and non-infectious conditions among ED patients with elevated PCT levels. The secondary aim was to assess the discriminatory accuracy of CRP and Lactate levels by analyzing their sensitivity, specificity, and overall predictive value for sepsis.

The cohort was intentionally restricted to patients with elevated PCT levels in order to reflect a clinically relevant subgroup in whom interpretation of elevated biomarkers (CRP, Lactate, and PCT) may be challenging.

Inclusion Criteria: Serum PCT level measured during presentation ≥ 0.51 ng/mL, age ≥ 18 years. Adequate and complete medical and laboratory data available to assess infection or sepsis status.

**Figure 1:**
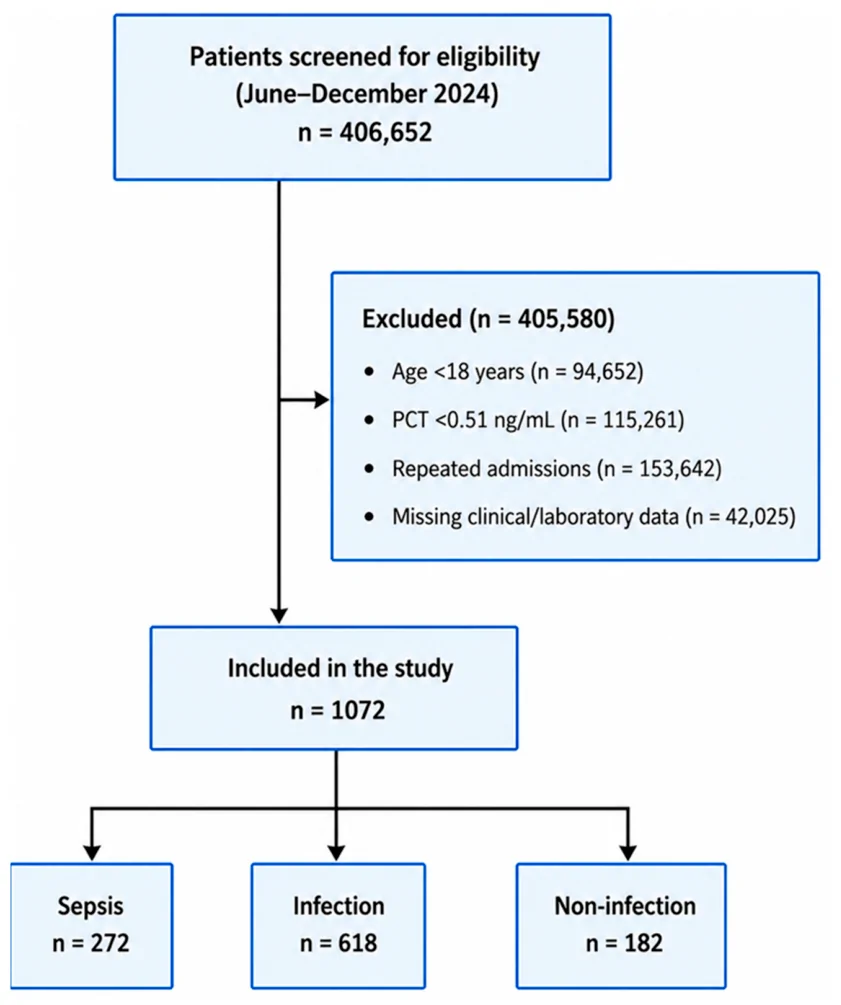
Flow diagram of patient selection and study group classification.

Patients younger than 18 years of age, those with serum PCT < 0.51 ng/mL, those with insufficient clinical, laboratory, or imaging data for reliable SOFA score calculation and study group classification, those with repeated hospital admissions during the same study period (only the first admission was included), and patients with incorrect or incomplete laboratory records due to pre-analytical or technical errors were excluded. The study selection process, including patient screening, exclusions, and final group allocation, is illustrated in Figure 1.

### 2.2. Clinical Definitions and Group Classification

All available clinical records, laboratory findings, microbiological results, imaging studies, consultation notes, hospitalization summaries, and follow-up data were independently reviewed by two physician investigators experienced in emergency and critical care medicine. The sepsis, infection, and non-infection groups had been established during routine clinical care before the study was conducted. During the retrospective review, investigators verified these classifications according to the Sepsis-3 criteria using the complete clinical records, microbiological findings, imaging studies, treatment course, and final discharge diagnoses rather than assigning new diagnoses based on biomarker values (PCT, CRP, or lactate). In cases of disagreement, consensus was achieved through joint re-evaluation.

Infection Group (IG): Patients were classified into the infection group if they had a clinically consistent focus of infection supported by physical examination, laboratory findings, and/or imaging studies (e.g., pneumonia, urinary tract infection, intra-abdominal infection, skin and soft tissue infection, catheter-related infection, cholecystitis, or central nervous system infection) but did not exhibit an increase of ≥2 points in the SOFA score above baseline.

Sepsis Group (SG): Patients who met the criteria for infection and exhibited evidence of infection-related organ dysfunction according to the Sepsis-3 definition were classified as the sepsis group (SOFA score ≥ 2 above baseline) [10]. Sepsis-3 criteria were applied retrospectively using SOFA scores calculated from the clinical and laboratory parameters recorded at the time of emergency department presentation. Baseline organ dysfunction related to chronic comorbidities (e.g., chronic kidney disease or chronic liver disease) was determined from available previous medical records whenever possible and was taken into account during case adjudication. Accordingly, chronic organ dysfunction itself was not considered evidence of sepsis-related organ dysfunction.

Non-Infection Group (NIG): Patients were assigned to the non-infection group if there were no clinical, laboratory, or radiological findings supporting an infectious etiology, and their clinical presentation was attributable to a non-infectious condition (e.g., acute kidney injury, gastrointestinal bleeding, electrolyte disturbance, vertigo, cerebrovascular disease, hypercalcemia, congestive heart failure, acute coronary syndrome, acute pancreatitis, malignancy-related processes, status epilepticus, pulmonary embolism, etc.). Patients were assigned to the non-infectious group only when no clinical, laboratory, microbiological, or radiological evidence of infection was identified during the index visit and subsequent clinical follow-up.

### 2.3. Statistical Analyses

Statistical analyses were performed using IBM SPSS Statistics for Windows, Version 27.0 (IBM Corp., Armonk, NY, USA). The distribution of continuous variables was assessed visually (histograms and probability plots) and analytically using the Shapiro–Wilk test. In descriptive analyses, means and standard deviations were used for normally distributed continuous variables, whereas medians and interquartile ranges (IQRs, 25–75%) were used for non-normally distributed variables. The Kruskal–Wallis test was utilized for comparisons among three groups. Categorical variables were analyzed using Pearson’s chi-square test or Fisher’s exact test, as appropriate. Receiver operating characteristic (ROC) curve analysis was performed to evaluate the discriminatory performance of PCT, CRP, and lactate for differentiating sepsis from infectious and non-infectious conditions. The area under the curve (AUC) with 95% confidence intervals (CIs) was calculated, and correlated AUCs were compared using DeLong’s test. Optimal cutoff values for PCT, CRP, and Lactate were determined using the Youden index (sensitivity + specificity − 1). At the determined cutoff points, sensitivity, specificity, positive predictive value (PPV), and negative predictive value (NPV) were calculated using 2 × 2 contingency tables. A two-sided *p* value < 0.05 was considered statistically significant. For post hoc pairwise comparisons following a significant Kruskal–Wallis test, Bonferroni correction was applied, and a *p* value < 0.016 was considered statistically significant.

The graphical abstract presented in this manuscript was generated using NotebookLM to assist with the conceptual visualization of the study’s key findings. The AI-generated output was subsequently reviewed, edited, and refined by the authors to ensure scientific accuracy, relevance, and compliance with journal guidelines. The authors take full responsibility for the accuracy, integrity, and originality of the graphical abstract and all content presented in this publication.

## 3. Results

A total of 1072 patients were included in the study. The median age of the cohort was 72 years (IQR, 62–81), and hypertension was the most common comorbidity (40.8%). The majority of patients required intensive care unit (ICU) admission (54.2%), and the overall mortality rate was 24.3%. The distribution of infection sources varied significantly across the study groups, with pneumonia and urinary tract infections being the most common sources (Table 1).

There were no significant differences among the groups in sex distribution, hemoglobin levels, or platelet counts. In contrast, BUN, creatinine, AST, ALT, CRP, lactate, and PCT levels differed significantly across the three groups (all *p* < 0.001; Table 2). Post hoc pairwise comparisons with Bonferroni correction demonstrated that CRP and PCT levels were significantly higher in the infection group than in the non-infection group (both *p* < 0.001), whereas lactate levels were significantly higher in the non-infection group (*p* = 0.013). The sepsis group had significantly higher CRP, lactate, and PCT levels than both the infection and non-infection groups (all *p* < 0.001, except lactate vs. non-infection, *p* = 0.008). Overall mortality differed significantly among the three groups (Pearson χ^2^ = 23.275, df = 2, *p* < 0.001). Post hoc pairwise comparisons with Bonferroni correction demonstrated that the infection group had significantly lower mortality than both the non-infection group (19.1% vs. 28.6%, *p* = 0.006) and the sepsis group (19.1% vs. 33.5%, *p* < 0.001), whereas mortality did not differ significantly between the non-infection and sepsis groups (28.6% vs. 33.5%, *p* = 0.272) (Table 2).

**Figure 2:**
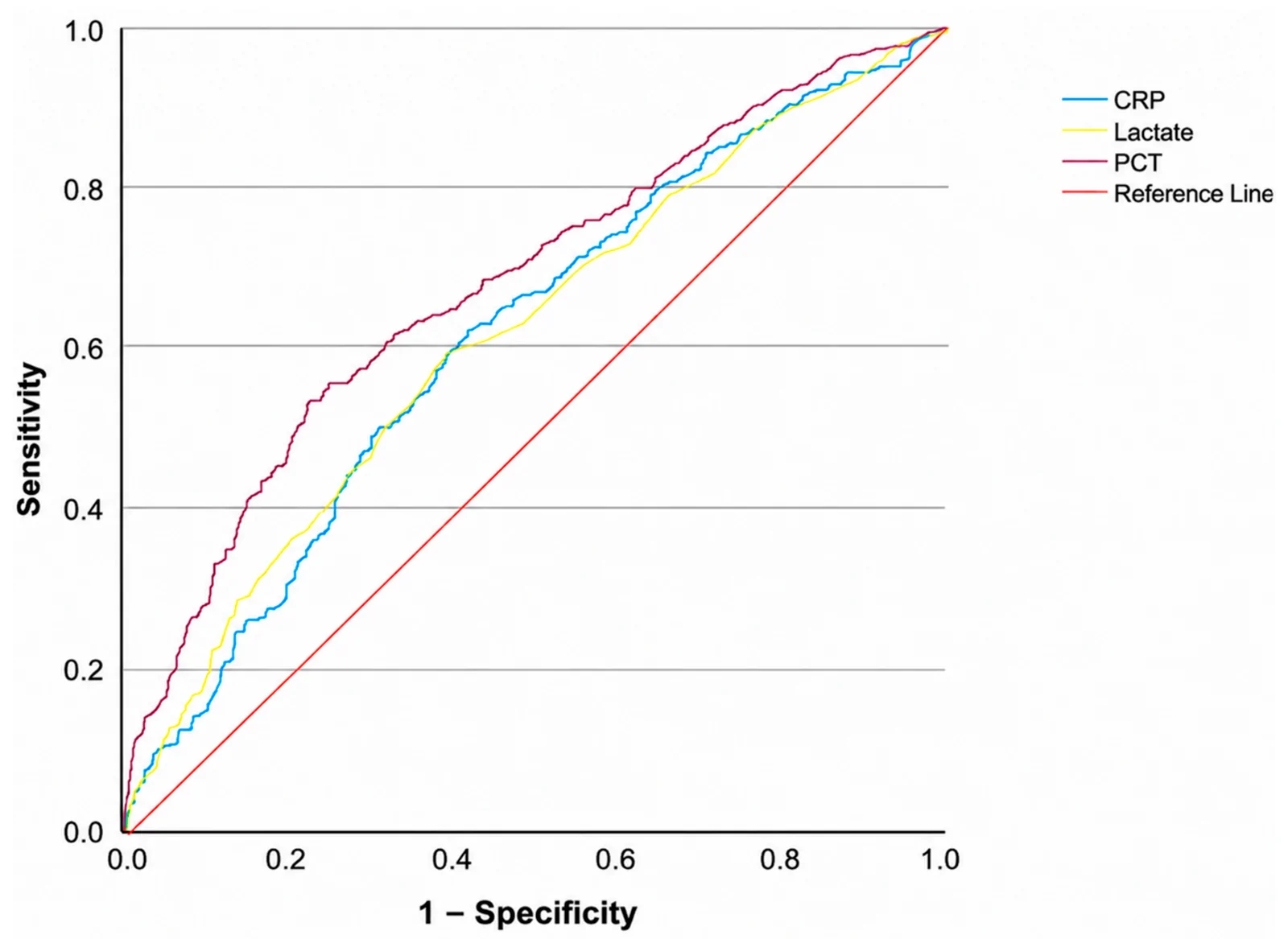
Receiver operating characteristic (ROC) curves of CRP, Lactate, and Procalcitonin for predicting Sepsis.

In our cohort, PCT, CRP, and lactate demonstrated different discriminatory performance for differentiating sepsis from infectious and non-infectious conditions. PCT showed the highest discriminatory performance, with an AUC of 0.683 (95% CI: 0.645–0.721; *p* < 0.001). The optimal cut-off determined by Youden’s index was 2.35 ng/mL, corresponding to a sensitivity of 65.8% and a specificity of 59.5%. CRP and lactate demonstrated comparable discriminatory performance, with AUCs of 0.619 (95% CI: 0.580–0.657; *p* < 0.001) and 0.620 (95% CI: 0.581–0.659; *p* < 0.001), respectively. The optimal cut-off values for CRP and lactate were 170 mg/L and 2.1 mmol/L, respectively. After Bonferroni correction for multiple comparisons (adjusted significance threshold: *p* < 0.016), the AUC of PCT remained significantly higher than that of CRP (*p* = 0.009). However, although PCT demonstrated a numerically higher AUC than lactate, this difference (*p* = 0.018) no longer met the adjusted significance threshold. No significant difference was observed between CRP and lactate (*p* = 0.974) (Table 3 and Table 4; Figure 2).

**Figure 3:**
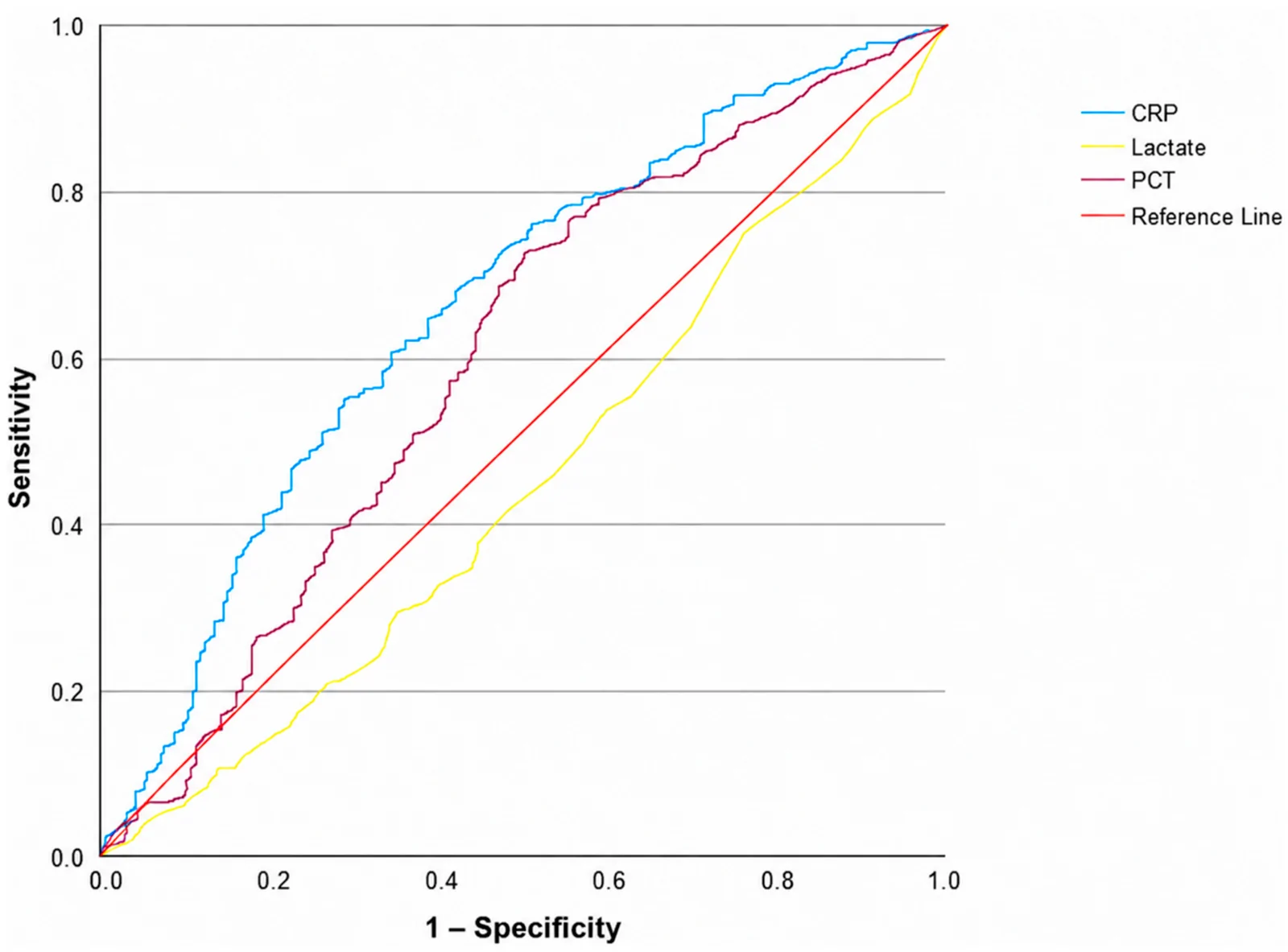
Receiver operating characteristic (ROC) curves of CRP, Lactate, and Procalcitonin (PCT) for predicting Infection.

For differentiating infection from non-infectious conditions, CRP showed modest discriminatory performance (AUC: 0.640, 95% CI: 0.592–0.687; *p* < 0.001), whereas PCT demonstrated limited discriminatory ability (AUC: 0.581, 95% CI: 0.530–0.631; *p* = 0.001). Lactate performed poorly, with an AUC of 0.439 (95% CI: 0.391–0.488; *p* = 0.013) (Table 3 and Figure 3).

**Figure 4:**
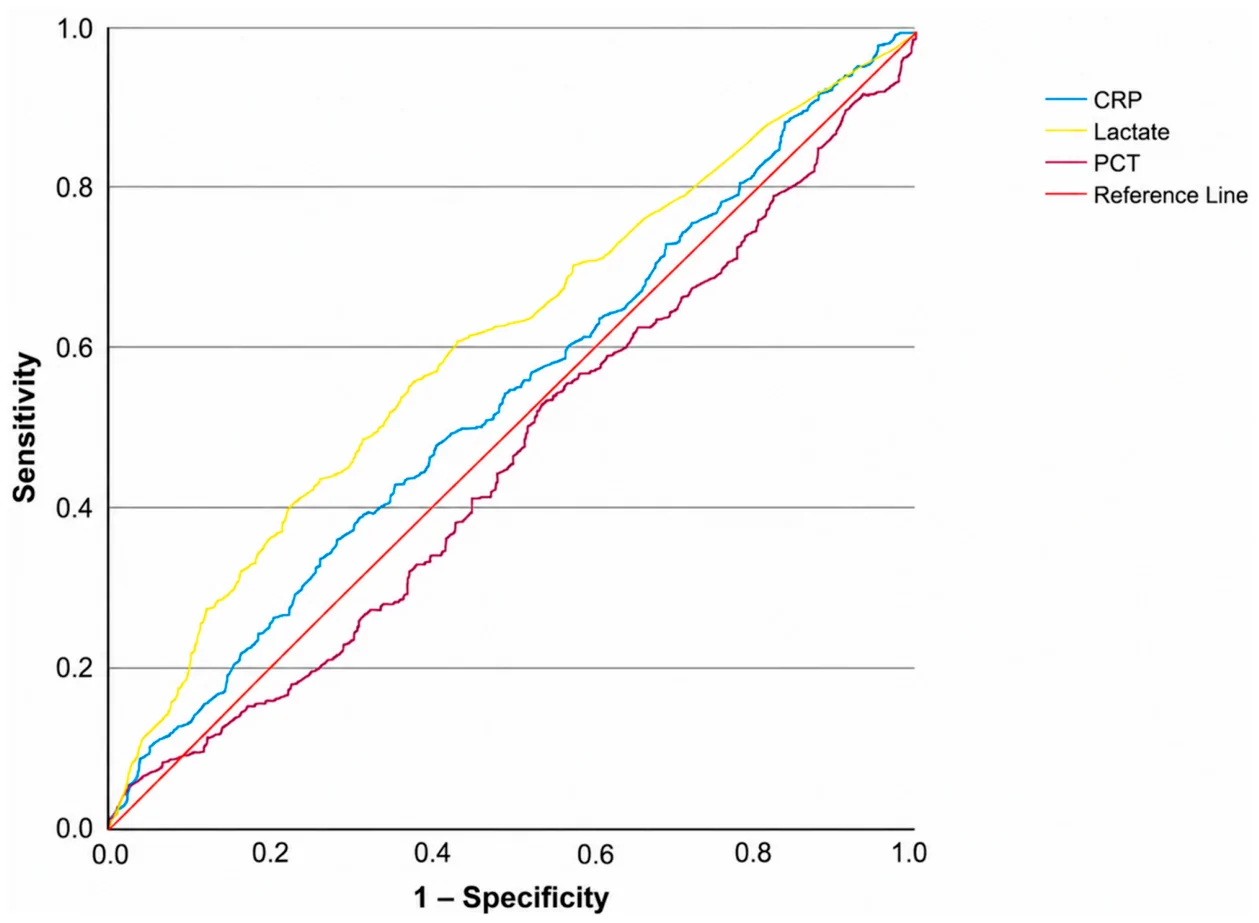
Receiver operating characteristic (ROC) curves of CRP, Lactate, and Procalcitonin (PCT) for predicting in-hospital mortality.

Additional ROC analyses were performed to evaluate the prognostic performance of the biomarkers for in-hospital mortality. Lactate demonstrated the highest discriminatory performance, with an AUC of 0.598 (95% CI: 0.558–0.639; *p* < 0.001). In contrast, CRP and PCT showed poor discriminatory performance, with AUCs of 0.533 (95% CI: 0.492–0.573; *p* = 0.11) and 0.470 (95% CI: 0.429–0.510; *p* = 0.13), respectively (Table 3 and Figure 4).

## 4. Discussion

In this single-center retrospective study of adults presenting with elevated PCT levels (≥0.51 ng/mL), we evaluated the diagnostic performance of Procalcitonin, CRP, and Lactate for differentiating sepsis from infectious and non-infectious conditions. Among the three biomarkers, PCT demonstrated significantly better discriminatory performance than CRP. Although PCT showed a numerically higher AUC than lactate, this difference did not remain statistically significant after correction for multiple comparisons. No significant difference was observed between CRP and lactate. Nevertheless, substantial overlap was observed among the study groups, indicating that biomarker measurements alone may not adequately distinguish sepsis from other infectious and non-infectious conditions. The low positive and relatively high negative likelihood ratios further indicate that none of the evaluated biomarkers provides sufficient diagnostic accuracy to establish or rule out sepsis when used in isolation. These findings are consistent with previous studies and support the concept that sepsis remains a clinical diagnosis [10]. As emphasized in the Sepsis-3 consensus, organ dysfunction assessed by the SOFA score, together with clinical evaluation, remains central to sepsis diagnosis. Therefore, all three biomarkers should be considered adjunctive tools rather than standalone diagnostic tests.

The moderate discriminatory capacity of PCT observed in our study aligns with several meta-analyses indicating that PCT is more useful for excluding bacterial infection than confirming it [11,12,13,14]. Simon et al. reported pooled sensitivity and specificity values of 76% and 69% for bacterial infection [1], which are slightly higher than our results but within a comparable range. The lower sensitivity and specificity in our cohort may relate to the inclusion of patients with elevated baseline PCT due to non-infectious etiologies, a phenomenon increasingly recognized in the literature [15,16,17,18].

CRP demonstrated modest discriminatory performance for identifying sepsis in our cohort, with an AUC comparable to that of lactate but significantly lower than that of PCT. In contrast, CRP showed the highest discriminatory performance for differentiating infectious from non-infectious conditions and also performed better than PCT for predicting in-hospital mortality, although its prognostic accuracy remained limited. These findings suggest that CRP may provide greater value in identifying the presence of an inflammatory or infectious process than in distinguishing sepsis from uncomplicated infection. The relatively high CRP cut-off identified in our study may be explained by the intentional inclusion of only patients with elevated baseline PCT (≥0.51 ng/mL). Consequently, the study population represented a clinically enriched cohort with a high inflammatory burden, likely shifting the optimal CRP threshold upward compared with studies conducted in unselected emergency department populations. Therefore, our findings should be interpreted within the context of this selected patient population rather than generalized to all emergency department patients.

Lactate is widely accepted as a prognostic marker in sepsis but has a more limited role in differentiating sepsis from other conditions in the ED setting. The modest discriminatory performance observed in our study (AUC 0.620) is consistent with previous reports suggesting that lactate primarily reflects global tissue hypoperfusion rather than infection-specific pathophysiology. This interpretation is further supported by two findings in our cohort. First, lactate demonstrated the highest prognostic performance for predicting in-hospital mortality among the evaluated biomarkers. Second, patients in the non-infection group had higher mortality than those in the infection group despite the absence of an infectious etiology. These findings suggest that elevated lactate concentrations are more closely associated with physiological derangement and illness severity than with the presence of infection itself. In addition, the study group definitions may have influenced the observed diagnostic performance of lactate. While the infection group comprised patients who did not meet the Sepsis-3 criterion of an acute increase of ≥2 points in the SOFA score above baseline, the non-infection group included patients with severe non-infectious conditions associated with organ dysfunction. Consequently, elevated lactate concentrations in the non-infection group may have contributed to its poor discriminatory performance for differentiating infectious from non-infectious conditions.

Our findings are also consistent with those of Ljungström et al., who reported moderate diagnostic performance for PCT (AUC 0.68) and more limited performance for CRP (AUC 0.57) and Lactate (AUC 0.57) in patients with suspected bacterial sepsis [19]. Although the study populations differed, both studies indicate that these biomarkers alone provide only moderate discrimination and should be interpreted within the clinical context.

An important methodological characteristic of this study is that all included patients already had elevated baseline PCT levels at ED presentation. Therefore, the present study should not be interpreted as an evaluation of the screening performance of PCT in an unselected emergency department population. Rather, it reflects a clinically enriched cohort designed to explore the interpretation and discriminatory behavior of inflammatory biomarkers in patients in whom elevated PCT levels had already raised diagnostic uncertainty. This design feature represents both a strength and a limitation of the study, as it allowed evaluation of biomarker performance in a clinically challenging subgroup while limiting the generalizability of the findings to unselected emergency department populations.

Several limitations should be acknowledged. First, the retrospective design precluded standardization of biomarker measurements in relation to symptom onset, disease progression, and treatment interventions, which may have influenced biomarker concentrations. Second, because only patients with elevated baseline PCT levels (≥0.51 ng/mL) were included, the study population represented a clinically enriched cohort. This design may have reduced biomarker variability, potentially attenuating the observed discriminatory performance of PCT and the other biomarkers. Furthermore, because positive and negative predictive values are prevalence-dependent, the PPV and NPV reported in this study may not be directly generalizable to unselected emergency department populations or settings with different sepsis prevalence. Third, microbiological confirmation was not available for all patients, and the classification of infectious versus non-infectious conditions may therefore have been affected by misclassification bias. Although all cases were independently reviewed by two experienced investigators and disagreements were resolved by consensus, formal inter-rater agreement statistics (e.g., Cohen’s κ) were not calculated. Fourth, because renal dysfunction contributes to the SOFA-based definition of sepsis and may influence PCT and lactate concentrations, part of the observed biomarker discrimination may reflect renal organ dysfunction rather than infection severity alone. Therefore, residual circularity and confounding by renal function cannot be completely excluded. Finally, because only patients with complete clinical and laboratory data were included, the analyses were based on complete-case data, which may have introduced selection bias.

## 5. Conclusions

In this clinically enriched cohort of emergency department patients with elevated baseline PCT levels, PCT showed better discriminatory performance for identifying sepsis than CRP and lactate, although the overall diagnostic accuracy of all three biomarkers remained modest. CRP demonstrated modest discrimination for differentiating infectious from non-infectious conditions, whereas lactate appeared to provide greater prognostic information for in-hospital mortality. Overall, the findings suggest that PCT, CRP, and lactate provide complementary clinical information rather than serving as standalone diagnostic tests. Their interpretation should always be integrated with clinical assessment and the Sepsis-3 criteria.

## Figures and Tables

**Table 1:** Descriptive Characteristics of the Study Population.

Variable	Value
Age median, IQR	72 (62–81)
Female *n* (%)	515 (48)
Group *n* (%)	Infection group 618 (57.6)
	Sepsis group 272 (25.4)
	Non-infection group 182 (17)
Comorbidities *n* (%)	Hypertension 437 (40.8)
	Diabetes mellitus 351 (32.7)
	Chronic kidney disease 241 (22.5)
	Coronary artery disease 215 (20.1)
	Solid malignancy 153 (14.3)
	COPD 100 (9.3)
ED termination *n* (%)	Discharged 165 (15.4)
	Ward 318 (29.7)
	ICU 581 (54.2)
	Exitus in ED 8 (0.7)
Source of infection *n* (%) ^&^	Pneumonia 339 (38.1)
	Urinary tract infection 295 (33.1)
	Bloodstream infection ^ 89 (10)
	Skin and soft tissue infection † 98 (11)
	Other * 108 (12.2)
In-hospital mortality *n* (%)	261 (24.3)

^ Catheter-related and peripheral bloodstream infections, † skin and soft tissue infection and diabetic foot infection, * intra-abdominal infection, cholangitis (biliary tract infection), central nervous system infection, septic arthritis; COPD: Chronic Obstructive Pulmonary Disease, ED: Emergency Department, ICU: Intensive Care; ^&^ some patients had multiple concurrent infection sources. Accordingly, 929 infection sources were identified among 890 patients with infection or sepsis; therefore, infection source categories are not mutually exclusive, and percentages may exceed 100% when summed.

**Table 2:** Baseline demographic, clinical, and laboratory characteristics of the study groups.

Variable	Sepsis *n* = 272	Infection *n* = 618	Non-Infection *n* = 182	*p*
Age	75 (65–83)	71 (62–79)	72 (59–80)	0.001
Female *n* (%)	146 (53.7)	282 (45.6)	87 (48.8)	0.086
In-hospital Mortality *n* (%)	91 (33.5)	118 (19.1)	52 (28.6)	<0.001
WBC × 10^3^/L	13.2 (8.7–18.8)	12.2 (8.1–17.3)	10.6 (6.9–15.5)	0.001
Neutrophil count × 10^3^/L	11.5 (7.3–16.6)	10.3 (6.4–14.9)	8.9 (5.3–12.7)	<0.001
Hemoglobin g/dL	11.3 (9.8–12.8)	10.9 (9.3–12.6)	10.9 (9.1–13)	0.11
Platelet count × 10^3^/mm^3^	194 (135–261)	199 (140–275)	197 (121–271)	0.44
BUN	43 (29–63)	30 (21–46)	39.5 (25–66)	<0.001
Creatinine, mg/dL	1.8 (1.1–3)	1.4 (0.9–2.39)	1.8 (1.2–3.1)	<0.001
AST, U/L	35 (18–75)	24 (16–41)	32 (19–67)	<0.001
ALT, U/L	21 (12–51)	16 (10–29)	21 (12–43)	<0.001
CRP, mg/L	206 (113–285)	153 (83–247)	95 (40–170)	<0.001
Lactate, mmol/L	2.7 (1.6–4.8)	1.9 (1.4–2.9)	2.2 (1.4–3.6)	<0.001
PCT ng/mL	6.4 (1.4–29.5)	1.8 (0.92–5.18)	1.1 (0.6–4.1)	<0.001

AST: aspartate aminotransferase, ALT: alanine aminotransferase, WBC: white blood cell count, BUN: Blood Urea Nitrogen. WBC, neutrophil count, platelet count, BUN, creatinine, AST, ALT, CRP, Lactate, PCT median and IQR.

**Table 3:** Diagnostic Performance of CRP, PCT, and Lactate for Identifying Infection, Sepsis and Mortality.

	AUC	Confidence Interval	*p*
Identification of Infection (Infection vs. Non-infection)			
CRP	0.640	0.592–0.687	*p* < 0.001
PCT	0.581	0.530–0.631	*p* = 0.001
Lactate	0.439	0.391–0.488	*p* = 0.013
Identification of Sepsis (Sepsis vs. Non-Sepsis)			
CRP	0.619	0.580–0.657	*p* < 0.001
PCT	0.683	0.645–0.721	*p* < 0.001
Lactate	0.620	0.581–0.659	*p* < 0.001
In-hospital mortality			
CRP	0.533	0.492–0.573	*p* = 0.11
PCT	0.470	0.429–0.510	*p* = 0.13
Lactate	0.598	0.558–0.639	*p* < 0.001

**Table 4:** Diagnostic performance of PCT, CRP, and Lactate for identifying sepsis based on ROC analysis and Youden index-derived cut-off values.

Variables	Cut Off	Specificity%	Sensitivity%	PPV%	NPV%	LR+	LR−	*p*
PCT ng/mL	2.35	59.5	65.8	35.4	83.7	1.62	0.57	<0.001
CRP mg/L	170	59.9	60.3	33.8	81.6	1.50	0.66	<0.001
Lactate mmol/L	2.1	51.7	63.2	30.8	80.5	1.31	0.71	<0.001

PPV: positive predictive value, NPV: negative predictive value, LR+: positive likelihood ratio, LR−: negative likelihood ratio.

## Data Availability

The datasets used and/or analyzed during the current study are available from the corresponding author on reasonable request.

## Abbreviations

The following abbreviations are used in this manuscript:

PCT	Procalcitonin
CRP	C-reactive protein
AUC	Area Under the Curve
WBC	White Blood Cell
STROBE	Strengthening the Reporting of Observational Studies in Epidemiology
ICU	Intensive Care Unit
IG	Infection Group
SG	Sepsis Group
NIG	Non-Infection Group
IQR	Interquartile Range
ROC	Receiver operating characteristic
CI	Confidence Interval
PPV	Positive Predictive Value
NPV	Negative Predictive Value
COPD	Chronic Obstructive Pulmonary Disease,
ED	Emergency Department
AST	Aspartate Aminotransferase
ALT	Alanine aminotransferase
BUN	Blood Urea Nitrogen
